# Early Interferon-γ Production in Human Lymphocyte Subsets in Response to Nontyphoidal *Salmonella* Demonstrates Inherent Capacity in Innate Cells

**DOI:** 10.1371/journal.pone.0013667

**Published:** 2010-10-27

**Authors:** Tonney S. Nyirenda, Anna E. Seeley, Wilson L. Mandala, Mark T. Drayson, Calman A. MacLennan

**Affiliations:** 1 Medical Research Council Centre for Immune Regulation and Clinical Immunology Service, Institute of Biomedical Research, School of Immunity and Infection, College of Medicine and Dental Sciences, University of Birmingham, Birmingham, United Kingdom; 2 Malawi-Liverpool-Wellcome Trust Clinical Research Programme, College of Medicine, University of Malawi, Blantyre, Malawi; 3 Department of Biochemistry, College of Medicine, University of Malawi, Blantyre, Malawi; 4 Division of Medical Microbiology, School of Infection and Host Defence, University of Liverpool, Liverpool, United Kingdom; New York University, United States of America

## Abstract

**Background:**

Nontyphoidal *Salmonellae* frequently cause life-threatening bacteremia in sub-Saharan Africa. Young children and HIV-infected adults are particularly susceptible. High case-fatality rates and increasing antibiotic resistance require new approaches to the management of this disease. Impaired cellular immunity caused by defects in the T helper 1 pathway lead to intracellular disease with *Salmonella* that can be countered by IFNγ administration. This report identifies the lymphocyte subsets that produce IFNγ early in *Salmonella* infection.

**Methodology:**

Intracellular cytokine staining was used to identify IFNγ production in blood lymphocyte subsets of ten healthy adults with antibodies to *Salmonella* (as evidence of immunity to *Salmonella*), in response to stimulation with live and heat-killed preparations of the D23580 invasive African isolate of *Salmonella* Typhimurium. The absolute number of IFNγ-producing cells in innate, innate-like and adaptive lymphocyte subpopulations was determined.

**Principal Findings:**

Early IFNγ production was found in the innate/innate-like lymphocyte subsets: γδ-T cells, NK cells and NK-like T cells. Significantly higher percentages of such cells produced IFNγ compared to adaptive αβ-T cells (Student's t test, *P*<0.001 and ≤0.02 for each innate subset compared, respectively, with CD4^+^- and CD8^+^-T cells). The absolute numbers of IFNγ-producing cells showed similar differences. The proportion of IFNγ-producing γδ-T cells, but not other lymphocytes, was significantly higher when stimulated with live compared with heat-killed bacteria (*P*<0.0001).

**Conclusion/Significance:**

Our findings indicate an inherent capacity of innate/innate-like lymphocyte subsets to produce IFNγ early in the response to *Salmonella* infection. This may serve to control intracellular infection and reduce the threat of extracellular spread of disease with bacteremia which becomes life-threatening in the absence of protective antibody. These innate cells may also help mitigate against the effect on IFNγ production of depletion of *Salmonella*-specific CD4^+^-T lymphocytes in HIV infection.

## Introduction

Nontyphoidal strains of *Salmonella* (NTS), in particular *Salmonella enterica* Typhimurium and Enteritidis (*S*. Typhimurium and *S*. Enteritidis) are a major cause of invasive disease, especially bacteremia and meningitis, in Africa [1], [2], [3], [4], [5], [6], [7], [8], [9], [10]. Children under two years of age [3], [6], [8] and HIV-infected adults [7], [9] are particularly susceptible and case-fatality rates for NTS bacteremia are high, being over 20% in children [6], [8] and up to 50% in adults [7]. A lack of typical clinical presentation for invasive NTS disease and affordable timely diagnostics [6], [8], [11], together with increasing levels of multi-drug resistance [4], [5], [11], [12] underlie the urgent need for new approaches to treat NTS.

In order to develop a vaccine against NTS, an improved understanding of the relevant modalities of protective immunity is required. We have previously shown the importance of antibody for complement-mediated cell-independent killing of NTS in the peripheral blood of young African children [3]. This protection can be lost in HIV-infected adults due to the presence of high titers of anti-LPS antibodies that block killing of *Salmonella* by antibodies against outer membrane proteins [13]. In addition to their capacity for extracellular survival, *Salmonellae* are facultative intracellular bacteria and their ability to survive within cells is essential for virulence [14]. The high susceptibility of individuals with chronic granulomatous disease, who lack normal phagocyte oxidative burst function, to infection with *Salmonellae* [15], [16] indicates the importance of cellular immune mechanisms against *Salmonella* in man. Therefore it is likely that control of intracellular infection moderates release of bacteria into body fluids and hence the dependence on antibodies against outer membrane proteins that protect against extracellular disease. Effective protection by a vaccine is likely to be achieved best by eliciting both these elements without inducing blocking antibodies. We have also recently demonstrated the importance of antibody acting as an opsonin for phagocyte cellular immunity against NTS in African children [17].

It is well established that IFNγ is a key cytokine for inducing mouse [18], [19], [20] and human [21], [22] macrophages to kill *Salmonella* through both oxidative burst and nonoxidative mechanisms. The importance of IFNγ in immunity to *Salmonella* in man is demonstrated by the heightened susceptibility of individuals with genetic deficiencies in the T helper 1 pathway, also known as the interleukin-(IL)12/23-interferon(IFNγ) axis, to recurrent infection with NTS [23], [24], [25], [26]. In those patients where the genetic defect is in the IL-12 or IL-12R genes [27], [28], [29] and in individuals with chronic granulomatous disease [30], subcutaneous IFNγ has successfully been used as an adjunct to antibiotic therapy in the treatment of invasive *Salmonella* disease. The presence of protective antibodies in these patients is the likely reason for the infections being manifest as local inflammatory lesions rather than fatal bacteremia or meningitis [24].

Additional support for an essential role for IFNγ in immunity to *Salmonella* comes from the mouse model of *Salmonella* infection. IFNγ-receptor-deficient mice are highly susceptible to attenuated *Salmonella* Typhimurium [31], as are mice that have been pretreated with antibodies to IFNγ [32], [33], [34], [35] or IL-12 [36]. Administration of recombinant IFNγ enhances killing of *Salmonella* in mice [32] and restores resistance to attenuated *Salmonella* after depletion of endogenous IL-12 using neutralizing anti-cytokine antibodies [36]. Blood levels of IFNγ increase both in *Salmonella* infections in mice [37], [38], [39], [40], [41] and in humans, particular in the case of systemic disease [42].

The course of untreated HIV infection is characterized by the loss of CD4^+^-T cells. Secretion of T helper 1 cytokines, in particular IFNγ, decreases during the course of HIV infection [43] and correlates with CD4 count [44]. There is downregulation of IFNγ production in both CD4^+^- and CD8^+^-T cell subsets [45] and a switch from a predominantly T helper 1 to a T helper 2 response [46]. *In vitro* infection of CD4^+^-T cells with HIV also results in downregulation of IFNγ expression [47], while commencement of HIV-infected patients on antiretroviral therapy leads to an increase in IFNγ production [48]. This reduction in IFNγ levels could, at least in part, account for the strong clinical association between invasive NTS disease and HIV/AIDS in Africa.

The clear importance for IFNγ in immunity to *Salmonella* and the ability of multiple lymphocyte subsets to produce this cytokine [24], led us to study IFNγ production in peripheral blood cells of healthy humans. We used intracellular cytokine staining (ICS) to identify the lymphocyte subsets that produce IFNγ when stimulated with NTS. We investigated lymphocytes in the innate arm of the immune system that do not use classical MHC-restricted antigen recognition (NK cells, γδ-T cells and NK-like T cells) as well as lymphocytes responsible for acquired cellular responses (CD4^+^- and CD8^+^-T cells). Identification of the cells that secrete this key cytokine will help in the design of new therapies against NTS.

## Materials and Methods

### Ethical Approval

Ethical approval for the use of anonymized blood samples in this study was granted by the Life and Health Sciences Ethical Review Committee of the University of Birmingham. Informed written consent was obtained from all participants.

### Blood samples

Anonymized blood samples were obtained from healthy adults (5 male:5 female; ages 21 to 41 years). Blood samples anticoagulated with sodium heparin at 4 IU/ml and EDTA (collected in EDTA-anticoagulated tubes, Becton Dickinson, UK) were used for intracellular cytokine staining (ICS) and conventional immunophenotyping respectively. Serum, separated and stored in aliquots at -80°C immediately after clotting, was used for studies of humoral immunity.

### *Salmonella*

The invasive Malawian *S*. Typhimurium isolate D23580 was used in all studies. Its genome has been sequenced at the Wellcome Trust Sanger Institute [12]. For experiments involving live *Salmonella*, D23580 was grown to log phase, washed twice with phosphate buffered saline (PBS) and added to heparin-anticoagulated whole blood or serum at 1/10^th^ of the final volume giving 10^6^ viable bacteria/ml. When required, the bacteria were heat-killed by incubating at 72°C for one hour. In the flow-cytometric antibody assay, D23580 in log growth phase was fixed with 1% formaldehyde in PBS. A whole-cell homogenate of D23580 was produced by disrupting washed D23580 using a Mini-Beadbeater (Biospec Products) as previously described [49]. This homogenate, at a final concentration of 10 µg/ml, was used to stimulate cells in heparinized-whole blood. Protein content of homogenates was determined by bicinchoninic acid (BCA) protein assay (Thermo Scientific).

### Intracellular cytokine staining

Half ml aliquots of heparin-anticoagulated blood were stimulated with *S*. Typhimurium D23580 within one hour of collection in 15 ml tubes at 37°C for six hours unless otherwise specified. At the time of stimulation, 5 µl CD28/CD49d co-stimulatory antibodies (Becton Dickinson) was added to each tube, including control tubes, in order to ensure that lack of costimulation did not limit the production of cytokines, in particular, by αβ-T cells. 10 µl Brefeldin A (Becton Dickinson) diluted 1∶10 with PBS was added to each tube after 2 hours. As positive control, blood was stimulated with phorbol 12-myristate 13-acetate (PMA) and ionomycin (both Sigma, UK) at final concentrations of 10 ng/ml and 1 µg/ml respectively. Negative control tubes were stimulated with PBS in place of *Salmonella*.

After the six hour stimulation period, adherent cells were resuspended by the addition of 50 µl EDTA (Becton Dickinson) to each tube. 50 µl aliquots of stimulated blood were incubated with a three-color panel of monoclonal antibodies (all Becton Dickinson) to the following cell-surface antigens in order to discriminate lymphocyte subsets and activated cells: PerCP-Cy5.5-conjugated CD4, CD8 or CD3 and APC-conjugated CD3, TCR-γδ or CD56, with PE-conjugated CD69 to indicate activated cells. Red cells were lysed with 10 volumes of 1X FACS Lysing Solution (Becton Dickinson) and samples washed with PBS 0.5% BSA prior to permeabilizing with 5 volumes of 1X FACS Permeabilizing Solution 2 (Becton Dickinson). Following a further wash with PBS 0.5% BSA, samples were incubated with the fourth color monoclonal antibody, FITC-conjugated anti-IFNγ antibody or an isotype control antibody followed by a final wash and resuspended in PBS 1% formaldehyde. Sample data were acquired and analyzed on a FACSCalibur flow cytometer using CellQuest Pro software (both Becton Dickinson). CD4^+^-T cells were defined as CD3^+^CD4^+^, CD8^+^-T cells as CD3^+^CD8^+^, γδ-T cells as CD3^+^TCRγδ^+^, NK cells as CD3^-^CD56^+^ and NK-like T cells as CD3^+^CD56^+^ lymphocytes.

### Immunophenotyping

Conventional immunophenotyping for lymphocyte subset analysis was performed on 50 µl samples of EDTA-anticoagulated blood as previously described using the following combinations of monoclonal antibodies (all Becton Dickinson): 1) TCRγδ-FITC, CD4-PE, CD8-PerCP-Cy5.5, CD3-APC; 2) CD56-PE, CD3-PerCP-Cy5.5, CD19-APC. Sample data were acquired and analyzed on a FACSCalibur flow cytometer using CellQuest Pro software. Tube 2 was also used for internal quality assurance to check that the sum of T, B and NK lymphocyte subset percentages was within 100±6%. Absolute lymphocyte subset counts were calculated as the product of the subset percentage and total lymphocyte count determined on a standard laboratory hematological analyzer.

### Monocyte activation assay

The ability of IFNγ, produced during the stimulation of whole blood with *Salmonella*, to activate monocytes, was assessed by stimulating fresh blood with plasma prepared from the whole blood stimulations and measuring CD38 expression on monocytes in these fresh blood samples. In order to allow the secretion of newly-synthesized IFNγ into the plasma, whole blood (or plasma, as control) was stimulated with *Salmonella* or PBS (negative control) as described above, but without the addition of Brefeldin A. After six hours, plasmas were collected by centrifugation and bacteria removed by sterilizing through 0.2 µm filters. 200 µl of each plasma was added to 500 µl fresh heparin-anticoagulated blood and incubated at 37°C for five hours. 50 µl samples of blood were labeled as described above, but using CD38-PE and CD14-APC antibodies. Monocytes were discriminated by CD14 against side-scatter histogram, and CD38 expression designated as the geometric mean fluorescent intensity (GMFI) in the FL2 histogram.

### Serum bactericidal assay and anti-*Salmonella* antibody assay

The serum bactericidal assay for the ability of serum to kill *S*. Typhimurium D23580 and flow cytometric assay for measurement of titer of anti-D23580 IgG and IgM were as previously described [3]. Briefly, viable D23580 in log growth phase at a 1/10^th^ volume was added to serum in a final volume of 100 µl and final concentration of 10^6^ cfu/ml. Killing of *Salmonella* at 37°C was measured over a three hour time course. For *Salmonella*-specific antibody determination, serum diluted 1∶10 with PBS was mixed with 1/10^th^ volume of formaldehyde-fixed D23580 at a final concentration of 2×10^8^ bacteria/ml, prior to detection of bound antibody with FITC-conjugated anti-human IgG and IgM (Sigma) and acquisition and analysis of data by flow cytometry.

### Statistical methods

Student's t test was used to compare percentages and absolute numbers of IFNγ-producing cells in different lymphocyte subsets. Log-transformation was used to normalize absolute cell counts within subsets.

## Results

### Detection of IFNγ-production by peripheral blood lymphocytes in response to stimulation with *Salmonella* using an optimized intracellular cytokine staining technique

**Figure 1:**
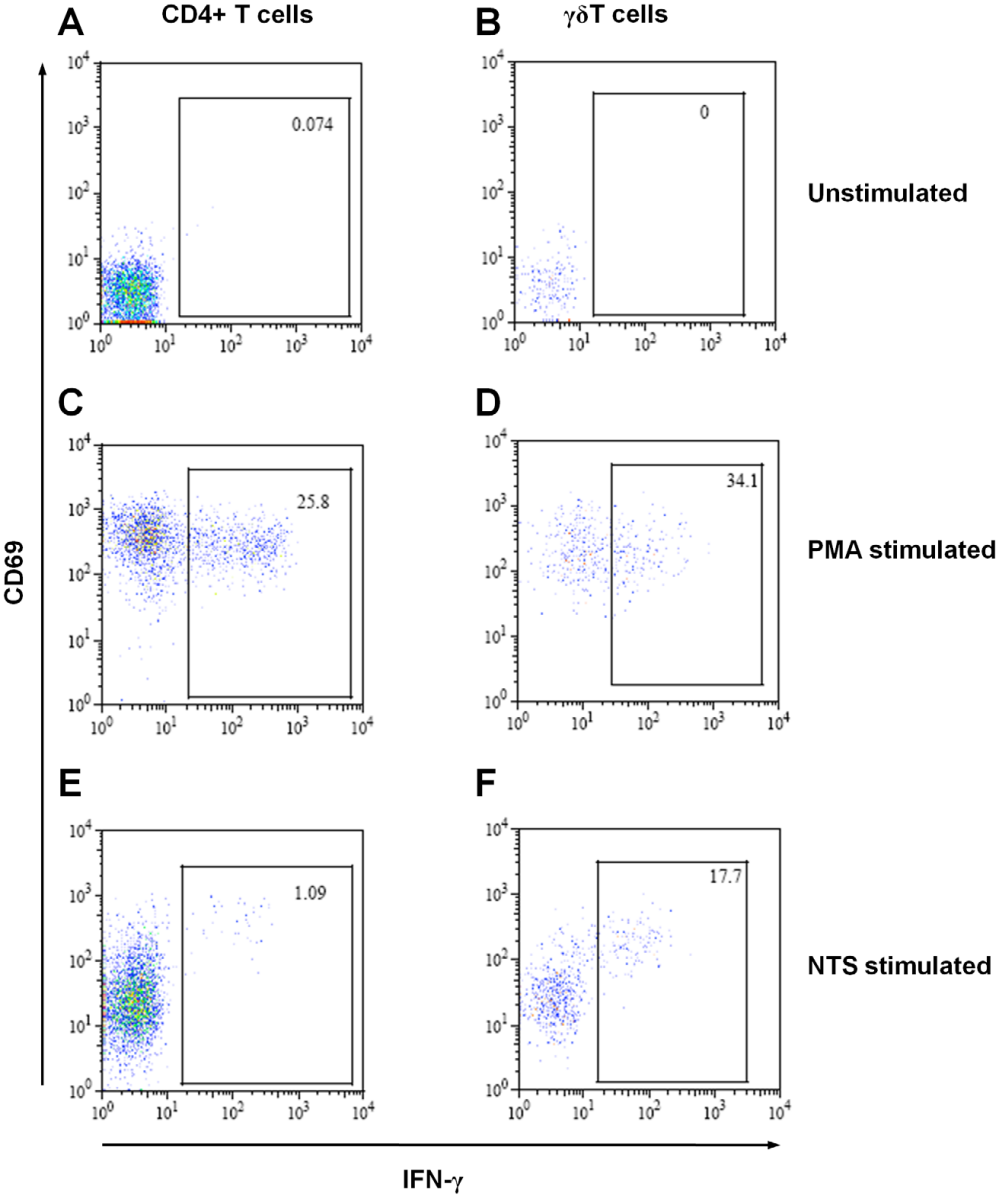
Detection of IFNγ-producing lymphocytes by flow cytometry following stimulation with *Salmonella*. Dot plots of CD4^+^-T cells (A, C and E) and γδ-T cells (B, D and F) from human peripheral blood from one healthy adult, either unstimulated (A and B), or stimulated with PMA (C and D) or *S*. Typhimurium D23580 homogenate (E and F), showing intracellular production of IFNγ and expression of CD69. Numbers within gates indicate percentage of IFNγ-producing cells. Each dot represents one cell.

We looked for IFNγ production by healthy adult peripheral blood lymphocytes stimulated with *S*. Typhimurium homogenate and PMA (Figure 1). Background numbers of IFNγ-producing cells were typically less than 0.1% (Figure 1A–B), while positive control PMA-stimulated cells gave responses of between 20% and 50% IFNγ-producing cells (Figure 1C–D). Cells producing IFNγ in response to stimulation with NTS also expressed the early activation antigen CD69, though not all CD69^+^ lymphocytes produced IFNγ (Figure 1E–F).

### Kinetics of IFNγ production by peripheral blood lymphocytes in response to stimulation with *Salmonella*

**Figure 2:**
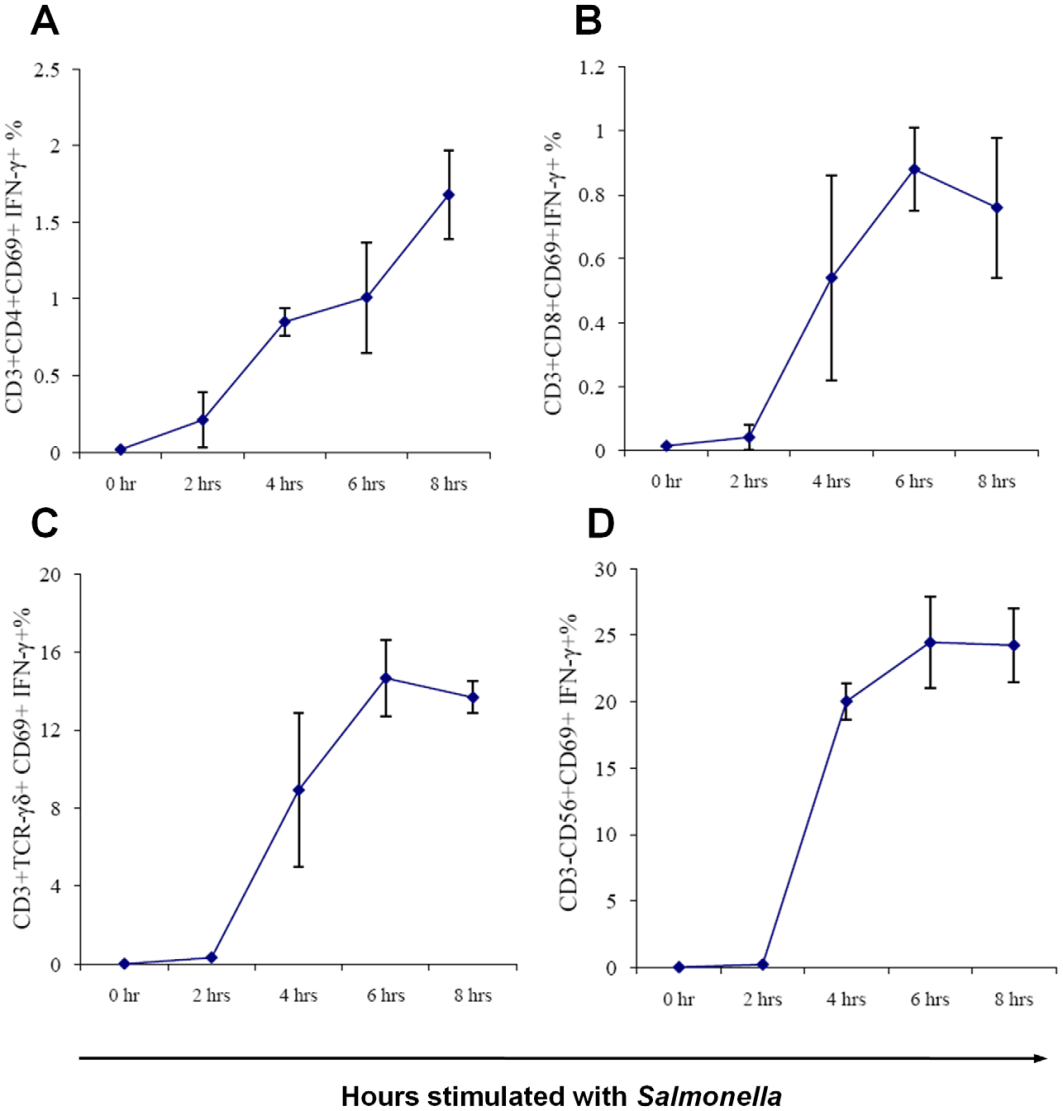
Kinetics of IFNγ production by peripheral blood lymphocytes in response to stimulation with *Salmonella*. Percentage of IFNγ-producing CD69^+^ (A) CD4^+^-T cells, (B) CD8^+^-T cells, (C) γδ-T cells and (D) NK cells over an eight hour time course following stimulation with *S*. Typhimurium D23580 homogenate added at 0 hours with addition of Brefeldin A at 2 hours. Data are mean ± sd of three experiments.

IFNγ production by different lymphocyte subsets was examined in relation to duration of stimulation with *Salmonella* homogenate in the blood of healthy adult donors using the ICS method described (Figure 2). Little, if any, cytokine production was detected after two hours of stimulation, though by four hours IFNγ production was present in cells of all four subsets examined: CD4^+^-, CD8^+^- and γδ-T cells, and NK cells. The percentage of IFNγ-producing cells plateaued at six hours for CD8^+^- and γδ-T cells, and NK cells (Figure 2B–D). By contrast, this percentage continued to increase over the next two hours for CD4^+^-T cells (Figure 2A). Based on these findings, we choose to stimulate blood with *Salmonella* for six hours in subsequent ICS assays. The percentage of IFNγ-producing cells was markedly higher in the γδ-T cells and NK cells subsets compared with CD4^+^- and CD8^+^-T cells.

### Immunity to *Salmonella* in peripheral blood from healthy adult donors

**Figure 3:**
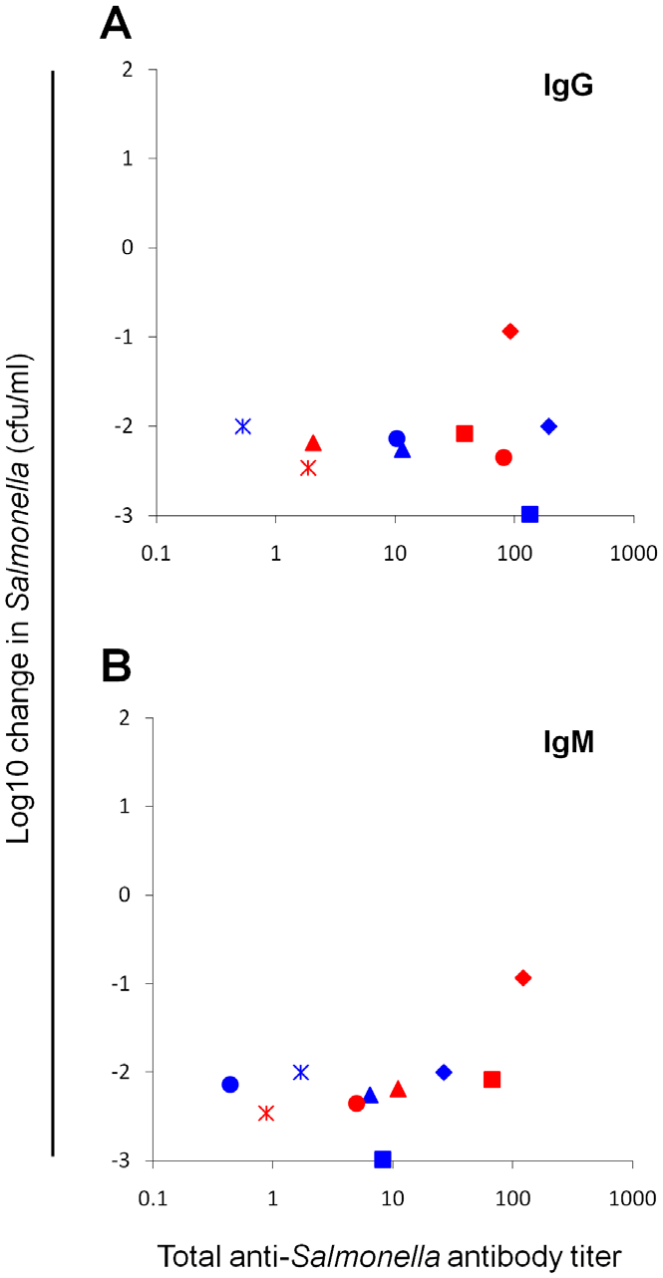
Anti-*Salmonella* antibody content and *Salmonella*-killing ability of serum from peripheral adult blood. (A) Anti-*S*. Typhimurium D23580 IgG and (B) anti-D23580 IgM titers determined by flow cytometry compared with *Salmonellae* D23580-killing ability determined by serum bactericidal assay at 180 minutes using serum prepared from peripheral blood from ten healthy adults. Negative values on y axis indicate reduction in viable *Salmonellae* with -1.0 corresponding to a 90% reduction in viable *Salmonellae* [3]. Each symbol corresponds to serum from one individual.

In order to provide an indication of the prior immunity and/or exposure to NTS in the ten anonymized healthy adult blood donors, we performed flow cytometric antibody assays to determine titers of IgG and IgM against *S*. Typhimurium isolate D23580 and serum bactericidal assays with D23580 (Figure 3). Sera from all ten donors contained anti-D23580 antibodies (>1.5 U) in the form of IgG or IgM or both classes of antibody. All sera were able to effect an approximately 90% or greater kill (1 log10 kill) of *Salmonella* in the serum bactericidal assay indicating that these anti-*Salmonella* antibodies are functional.

### IFNγ-production by peripheral blood lymphocyte subsets in response to stimulation with *Salmonella*

**Figure 4:**
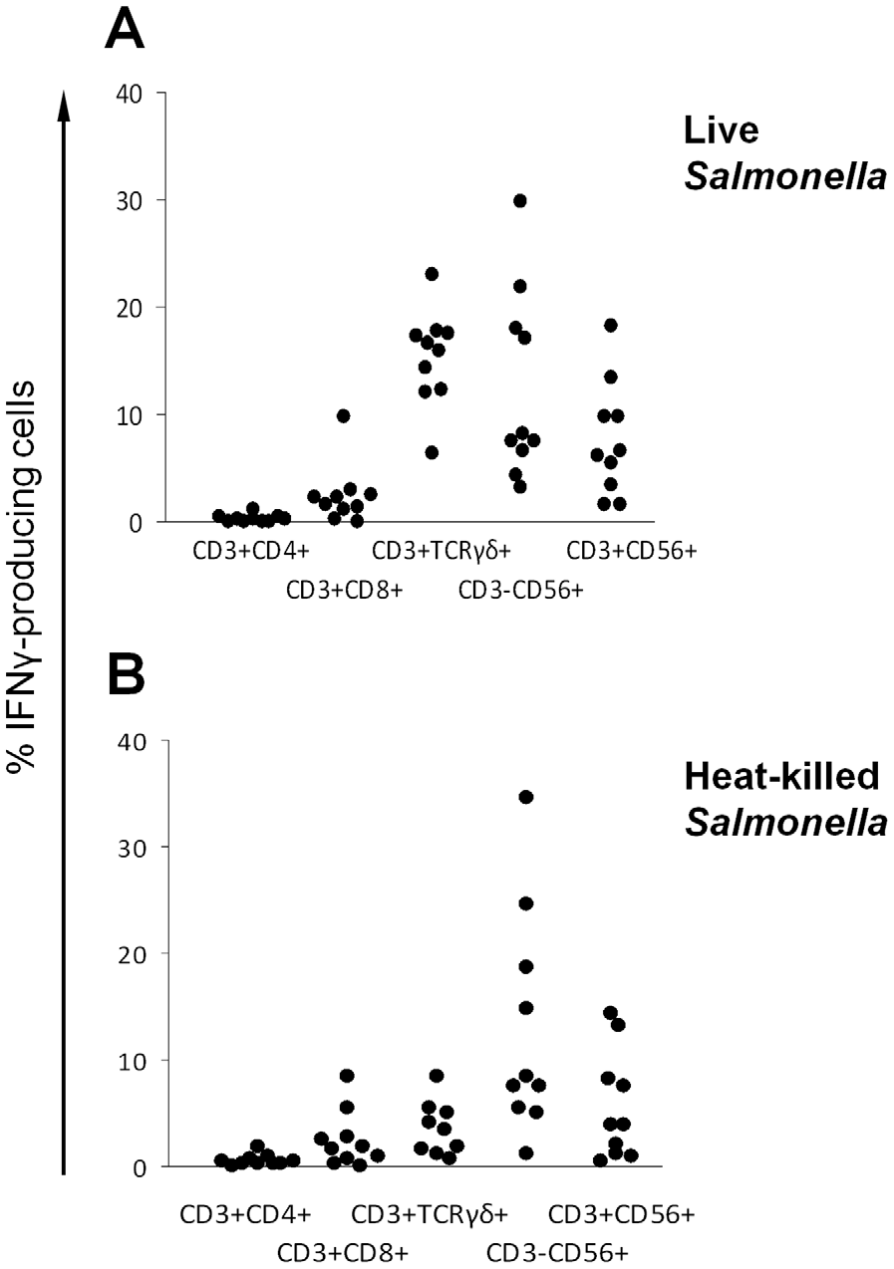
IFNγ production by lymphocyte subsets in response to stimulation with live and heat-killed *Salmonella*. Percentages of IFNγ-producing lymphocyte subsets in peripheral blood from ten healthy adults following 6 hours of stimulation with (A) live and (B) heat-killed *S*. Typhimurium D23580. Each dot corresponds to the percentages of IFNγ-producing cells in each lymphocyte subset in blood from one individual. CD3^+^CD4^+^: CD4^+^-T cells; CD3^+^CD8^+^: CD8^+^-T cells; CD3^+^TCRγδ^+^: γδ-T cells; CD3^−^CD56^+^: NK cells; CD3^+^CD56^+^: NK-like T cells.

Next we investigated the response of blood lymphocytes from the ten healthy adult donors to stimulation with live *S*. Typhimurium D23580 for six hours (Figure 4A). Over this time course, significantly higher percentages of the innate/innate-like lymphocyte subsets (NK cells, NK-like T cells and γδ-T cells) produced IFNγ compared with the adaptive T cell subsets (CD4^+^- and CD8^+^-T cells), despite evidence of prior exposure to *Salmonella* in these donors from the studies of their humoral immune response. Using Student's t test, *P*<0.001 was obtained for difference in percentage of IFNγ-producing cells in each innate and innate-like lymphocyte subset compared with percentage of IFNγ-producing CD4^+^-T cells. *P*≤0.02 was obtained for difference in percentage of IFNγ-producing cells in each innate/innate-like lymphocyte subsets compared with CD8^+^-T cells.

### IFNγ-production by peripheral blood lymphocyte subsets in response to stimulation with live compared with heat-killed *Salmonella*

Previous reports indicate a difference in immune response to *Salmonella* depending on whether the bacteria are alive or dead [39], [50], [51]. To assess this in the context of the current study, IFNγ production by lymphocyte subsets stimulated with heat-killed D23580 was compared to that by cells stimulated with live D23580 (Figure 4B). No significant difference was found in the percentage of IFNγ-producing cells in each lymphocyte subset (t test, *P*>0.2), except for γδ-T cells where markedly reduced IFNγ-producing cells were generated by stimulation with dead bacteria (t test, *P*<0.0001).

### Ability of IFNγ produced by peripheral blood lymphocytes to stimulate blood monocytes

**Figure 5:**
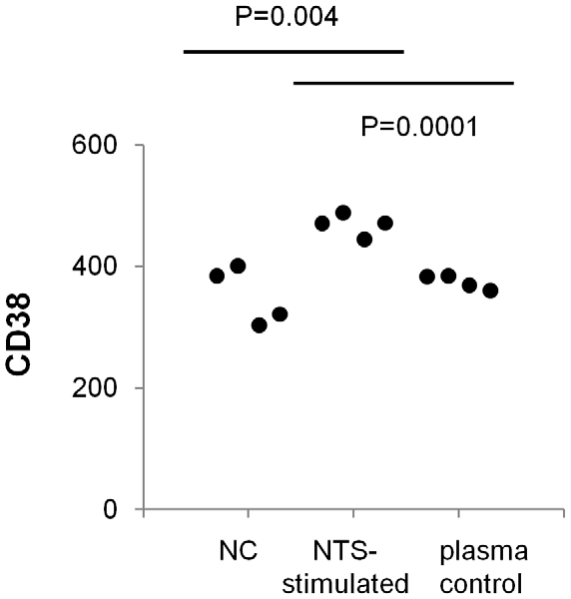
Effect of plasma from *Salmonella* stimulation of whole peripheral blood on monocyte CD38 expression. Plasma from *S*. Typhimurium-stimulated whole blood (NTS stimulated) was separated and filter-sterilized prior to adding to fresh blood for five hours followed by measurement of CD38 expression on monocytes. CD38 expression was significantly higher than for blood incubated with plasma taken from unstimulated blood (NC) or for blood incubated with plasma stimulated with NTS in the absence of cells (plasma control). *P* values are from Student's t test. Data are from four experiments.

In order to give an indication of whether IFNγ produced by peripheral blood lymphocytes in the ICS assays was functional, we measured the ability of plasmas from whole blood stimulated with *Salmonella* to activate monocytes. CD38 was chosen as an indicator of monocyte activation, because it is strongly upregulated on human monocytes by IFNγ, but not by other monocyte-activating factors, including other cytokines such as TNFα and GM-CSF, and bacterial components such as LPS [52]. Surface expression of CD38 on monocytes in four blood samples was measured by flow cytometry following 5 hours of stimulation with plasmas derived from *Salmonella*-whole blood stimulation assays. Mean GMFI expression of CD38 was 469 compared with 352 for monocytes from blood samples stimulated with plasma from unstimulated blood (negative control) (*P* = 0.004) (Figure 5).

In order to ensure that raised CD38 expression was not due to direct bacterial stimulation, plasmas from whole blood stimulation assays were sterilized through 0.2 µm filters before adding to fresh blood samples. To confirm that bacterial components such as LPS in the plasmas were not the cause of raised monocyte CD38 expression, we also stimulated fresh whole blood with plasma samples that had been separated from cells prior to incubating with *Salmonella* for 6 hours (plasma control). Mean CD38 expression on monocytes from these samples was 374 and was significantly lower than observed with blood stimulated by plasmas collected from whole blood stimulated with *Salmonella* (P = 0.0001), but was no different to CD38 expression on monocytes in blood incubated with plasma from unstimulated blood (Figure 5).

### Absolute numbers of IFNγ-producing lymphocytes in peripheral blood in response to stimulation with *Salmonella*

While our experiments so far show the proportion of lymphocytes in each subset producing IFNγ, we wanted to determine the absolute numbers of IFNγ-producing cells for each lymphocyte subset in peripheral blood. To do this, we first measured the absolute numbers of each lymphocyte subset, calculated from the percentage of each subset within the lymphocyte population by conventional immunophenotyping and the total lymphocyte count from the hematological analyzer. Then, the absolute numbers of IFNγ-producing lymphocytes was assessed as the product of each absolute count and percentage IFNγ-producing cells (Table 1).

**Table 1:** Percentage and absolute numbers of IFNγ-producing cells in different lymphocyte subsets in peripheral blood from ten healthy adults following stimulation with *Salmonella*.

	CD4^+^-T cells			CD8^+^-T cells			γδ-T cells			NK cells			NK-like T cells			IFNγ+ Cell Count		
	(CD3+CD4+)			(CD3+CD8+)			(CD3+TCRγδ+)			(CD3−CD56+)			(CD3+CD56+)					
Donor	Count	%IFNγ+	IFNγ+Count	Count	%IFNγ+	IFNγ+Count	Count	%IFNγ+	IFNγ+Count	Count	%IFNγ+	IFNγ+Count	Count	%IFNγ+	IFNγ+Count	Acqlymp	Innatelymp	Totallymph
**1**	957	1.08	10	805	2.29	18	143	14.3	20	157	29.68	47	90.4	3.1	3	**29**	**70**	**99**
**2**	733	0.36	3	527	9.66	51	144	17.27	25	632	28.35	179	110	18.27	20	**54**	**224**	**278**
**3**	671	0.36	2	367	0.26	1	54	6.30	3	104	7.46	8	57.90	1.70	1	**3**	**12**	**16**
**4**	651	0.18	1	387	0.00	0	372	17.42	65	246	4.30	11	55.90	6.18	3	**1**	**79**	**80**
**5**	544	0.00	0	486	3.05	15	126	11.98	15	122	8.25	10	135	9.72	13	**15**	**38**	**53**
**6**	829	0.03	0	593	2.28	14	76.1	16.68	13	258	16.99	44	62.80	9.83	6	**14**	**63**	**76**
**7**	583	0.00	0	424	1.10	5	66.5	22.89	15	334	7.40	25	47.50	5.44	3	**5**	**42**	**47**
**8**	502	0.13	1	399	1.46	6	102	12.21	12	375	6.69	25	592	6.64	39	**6**	**77**	**83**
**9**	1200	0.36	4	612	0.26	2	94.2	6.3	6	355	7.46	27	155	13.45	21	**6**	**53**	**59**
**10**	591	0.18	1	244	0.00	0	50.9	17.42	9	175	4.30	8	42.1	1.70	1	**1**	**17**	**18**
**Geometric Mean**		**0.22**	**1**		**0.84**	**3**		**14.30**	**14**		**12.1**	**25**		**7.60**	**5**	**7**	**50**	**60**

For each lymphocyte subset in the peripheral blood of each subject, the absolute concentration of cells (Count; cells/µl), percentage of IFNγ-producing cells (%IFNγ^+^) and absolute numbers of IFNγ-producing cells (IFNγ^+^ Count; cells/µl) following stimulation for six hours with live *S*. Typhimurium D23580 were determined by intracellular cytokine staining and a hematological analyzer. All adaptive lymphocytes (Acq lymph; CD4^+^-and CD8^+^-T cells), innate lymphocytes (Innate lymp; γδ-T cells, NK cells and NK-like T cells), together with total numbers of IFNγ-producing lymphocytes are shown in the right-hand columns. Geometric means are for average percentages and absolute numbers of cells across all subjects.

Geometric mean absolute counts for IFNγ-producing cells were approximately 10-fold lower for CD4^+^-T cells (1 cell/µl) and CD8^+^-T cells (3 cells/µl) compared with the innate/innate-like subsets (γδ-T cells 14 cell/µl, NK cells 25 cells/µl, NK-like T cells 5 cells/µl). Although a wide range of total IFNγ-producing lymphocytes (16 to 278 cells/µl) was found in peripheral blood across the ten donors, absolute numbers of IFNγ-producing lymphocytes in innate/innate-like subsets were significantly higher than those in the adaptive subsets (geometric means: 50 compared with 7 cells/µl, t test *P*<0.001).

## Discussion

In the present study, we have used intracellular cytokine staining to examine the production of IFNγ by different lymphocyte subsets in response to stimulation with *S*. Typhimurium D23580, a well-characterized invasive African strain of NTS [12]. Having previously established an importance for antibody in protecting against NTS in Africans [3], [17], we wanted to investigate the cellular arm of the immune response to *Salmonella* in man. Since T helper 1 cell immunity is key for protection against invasive *Salmonella* disease [24] and IFNγ is the pre-eminent effector T helper 1 cytokine, we focused our attention on production of this cytokine.

Our choice of studying cytokine production in *ex vivo* peripheral blood was partly pragmatic, in view of the limited scope for *in vivo* studies of infection in man. Since the commonest presentation of invasive NTS disease in Africans is bacteremia, *in vitro* study of infection in peripheral blood arguably provides a reasonable model of this clinical problem. It could also be argued that a principal requirement for IFNγ in *Salmonella* infection is within the secondary lymphoid tissues, particularly in the context of recurrent intracellular infection observed in individuals with genetic T helper 1 pathway deficiencies. Investigation of IFNγ levels in man in such tissues would be difficult to conduct. The relatively short duration of the experimental model (hours as opposed to days) reflects the rapid time-course of invasive NTS disease *in vivo*. Approximately half of all African children who die from acute NTS bacteremia do so within the first 24 to 48 hours of admission (unpublished findings). This is similar to the length of time required for a positive blood culture result where facilities for this exist.

Previous studies on cytokine production in response to *Salmonella* infection have tended to focus on a limited number of lymphocyte subsets and few studies have been performed on human tissue. We wanted to understand the immune response of a broader range of lymphocyte subsets. In particular, we sought to examine whether there were differences in responses between the innate/innate-like subsets compared to lymphocyte subsets of the acquired immune response (the TCR-αβ lymphocytes). Further subdivision of these subsets could be performed with a flow cytometer equipped with more than four fluorescence detector channels. Nevertheless, the panel of markers used in this study permitted us to differentiate the principle subsets of the T and NK lymphocyte lineages and to distinguish between innate and adaptive cells. Since lymphocyte subset sizes can vary considerably between individuals [53], we also wanted to determine absolute as well as relative numbers of IFNγ-producing cells in response to stimulation with *Salmonella*.

The principle finding of this study is the high inherent capacity within the innate immune system for IFNγ production relative to the adaptive immune system in response to NTS, even when peripheral blood from adults with evidence of existing immunity and/or prior exposure to *Salmonella* is examined. This latter point is important, since it reduces the possibility that the relative lack of acquired lymphocytes responsive to *Salmonella* was the result of immune naivety towards *Salmonella*, though higher levels of *Salmonella*-specific αβ-T lymphocytes would be expected among Africans where exposure to *Salmonella* is likely to be much higher.

In relation to percentages of cells in each subset that produce IFNγ, γδ-T cells, NK cells and NK-like T cells had significantly higher percentages of IFNγ-producing cells than CD4^+^- and CD8^+^-T cells. Given their smaller overall sizes, it is even more striking that when absolute cell counts are examined, the numbers of IFNγ-producing innate/innate-like subsets are still dominant. Geometric mean values for IFNγ-producing cell counts in the innate/innate-like subsets (14, 25 and 8 cells/µl) are higher than those for the classical adaptive subsets (1 and 3 cells/µl), with a 7-fold overall difference in IFNγ-producing cells between the two broad categories (7 cells/µl for adaptive lymphocytes and 50 cells/µl for innate/innate-like cells).

Whilst our understanding of *Salmonella* infections in individuals with genetic deficiencies of the T helper 1 pathway demonstrates the importance of T helper 1 immunity and IFNγ for protection against *Salmonella*, it does not indicate which of the IFNγ-producing cell subsets are needed for this protection. Our finding of capacity within the innate system for IFNγ production suggests that during acute infections with *Salmonella*, this capacity may suffice and priming of the acquired T cell response through prior exposure/infection or vaccination may not be essential. The functionality of the secreted IFNγ demonstrated by the upregulation of CD38 expression on blood monocytes adds further significance to these observations.

Using the mouse model of *Salmonella* infection, IFNγ production has been demonstrated in a variety of individual lymphocyte subsets. A strong T helper 1 response is characteristic of *S*. Typhimurium infection in the mouse, with a large expansion in numbers of activated IFNγ-secreting CD4^+^- and CD8^+^-T cells in the blood, though this response takes a week to develop during primary infection [54], [55], [56]. However, splenic cells from mice with severe combined immunodeficiency (SCID) that lack T cells produce elevated levels of IFNγ after stimulation with *S*. Typhimurium [57], suggesting that NK or other innate cells are responsible for IFNγ production. NK cell numbers are elevated post infection with *Salmonella* [58], [59], [60] and are an early source of IFNγ [59] and mice depleted of NK cells show diminished resistance to *Salmonella* infection [58], [61]. Human NK cells have also been shown to produce IFNγ *in vitro* in response to stimulation with *Salmonella* and to clear macrophages of *Salmonella* infection [61].

An important subset in the innate response to NTS is the γδ-T cell subset. Since these cells can produce both innate and adaptive responses, it is probably more accurate to describe them as ‘innate-like’, since although in the current study they are most likely mounting an innate response, they have the capacity to respond specifically through their T cell receptors. Expansion of the γδ-T cell subset in peripheral blood has been found during *Salmonella* infection in man, particularly with systemic disease [62], as has enlargement of the NK-like T cell subset [63]. γδ-T cells from patients with *Salmonella* infection produce high levels of IFNγ [42]. Likewise, increased numbers of γδ-T cells are found in murine salmonellosis [60], [64], [65], [66], particularly in the peritoneal cavity, and these produce IFNγ in response to *Salmonella*-infected macrophages. Depletion of γδ-T cells in the mouse results in increased susceptibility to *Salmonella* infection [67].

While the response of other subsets investigated was independent of whether live or dead bacteria were used for stimulation, it is intriguing that a much higher proportion of γδ-T cells produced IFNγ in response to live *Salmonella* compared with heat-killed *Salmonella*. The necessity of live *Salmonella* for induction of a T helper 1 response in the mouse has previously been described [39], [50], [68] and attributed to better IL-12 induction by live bacteria [51]. Our finding in relation to IFNγ production by γδ-T cells in man suggests that the γδ-T cell subset may have an important role in protecting individuals from acute *Salmonella* infections.

IFNγ production by the innate arm of the immune system produces a rapid response that may be key in controlling the early stages of infection. This, by itself, may not always be sufficient to clear *Salmonella* infection in man and prevention of persistent infection in the macrophage beds that can lead to recrudescence of infection. Studies in the mouse indicate that αβ-T cells are not required during the first week of *Salmonella* infection [69], [70] and IFNγ is produced in response to IL12 and IL18 in *rag* ^−/−^ and SCID mice that lack T cells [71]. These findings all support the concept that cells other than αβ-T cells are responsible for production of the IFNγ required for survival in the first week of *Salmonella* infection. Nevertheless, mice lacking αβ-T cells fail to clear *Salmonella* and die later during *Salmonella* infection [31], [70], [72], [73], [74], [75]. Surprisingly, this clearance does not appear to involve IFNγ, since depletion of this cytokine from day 6 of *Salmonella* infection in the mouse does not prevent bacterial clearance [76]. This raises the possibility that in many instances IFNγ-production by αβ-T cells is not required for immunity to *Salmonella*. In contrast, IL-2 has been found to enhance clearance of *Salmonella* in the mouse raising the possibility that this cytokine is more important than IFNγ during the late response to *Salmonella* infection [77].

Our study has potential implications in relation to the reduction of IFNγ levels seen in HIV infection and the high susceptibility of Africans living with HIV/AIDS to NTS bacteremia. Since HIV targets cells that express CD4, especially CD4^+^-T cells, and decreased IFNγ production in the context of HIV infection occurs in cells of the acquired immune system [45], [47] the finding of inherent capacity for IFNγ production among innate cells could be exploited to therapeutic advantage. Expansion of non-CD4-expressing cells of the innate immune system with IFNγ-producing capacity, in particular NK cells and γδ-T cells, could compensate for lost IFNγ production by CD4^+^-T cells, particularly in advanced HIV/AIDS. This could be used both in the prevention and treatment of invasive NTS disease in such individuals.

Our present findings indicate that the innate cellular arm of the immune system has an inherent capacity for production of IFNγ. Taken in the context of other work on the role of antibody in protection against NTS bacteremia, these findings suggest that the primary requirement of a vaccine against NTS should be the induction of a protective antibody response against these bacteria. Without such antibody, serum bactericidal function [3] and the opsonic activity required for phagocyte cell function [17] are both abrogated leaving children vulnerable to both extracellular and intracellular growth of *Salmonella*. Prevention of persistent *Salmonella* infection with recrudescent disease, as commonly found in HIV-infected Africans [7], is also an important goal of vaccination and this may require the development of a robust adaptive cellular response to NTS. Further insight into the need for this will be gained through the study of at-risk populations where the disease is most prevalent: young children and HIV-infected adults in sub-Saharan Africa.
